# DMRT1 Is Required for Mouse Spermatogonial Stem Cell Maintenance and Replenishment

**DOI:** 10.1371/journal.pgen.1006293

**Published:** 2016-09-01

**Authors:** Teng Zhang, Jon Oatley, Vivian J. Bardwell, David Zarkower

**Affiliations:** 1 Developmental Biology Center and Department of Genetics, Cell Biology, and Development, University of Minnesota, Minneapolis, Minnesota, United States of America; 2 School of Molecular Biosciences, Center for Reproductive Biology, College of Veterinary Medicine, Washington State University, Pullman, Washington, United States of America; 3 University of Minnesota Masonic Cancer Center, Minneapolis, Minnesota, United States of America; Cornell University, UNITED STATES

## Abstract

Male mammals produce sperm for most of postnatal life and therefore require a robust germ line stem cell system, with precise balance between self-renewal and differentiation. Prior work established *doublesex-* and *mab-3*-related transcription factor 1 (*Dmrt1*) as a conserved transcriptional regulator of male sexual differentiation. Here we investigate the role of *Dmrt1* in mouse spermatogonial stem cell (SSC) homeostasis. We find that *Dmrt1* maintains SSCs during steady state spermatogenesis, where it regulates expression of *Plzf*, another transcription factor required for SSC maintenance. We also find that *Dmrt1* is required for recovery of spermatogenesis after germ cell depletion. Committed progenitor cells expressing *Ngn3* normally do not contribute to SSCs marked by the *Id4-Gfp* transgene, but do so when spermatogonia are chemically depleted using busulfan. Removal of *Dmrt1* from *Ngn3*-positive germ cells blocks the replenishment of Id4-GFP-positive SSCs and recovery of spermatogenesis after busulfan treatment. Our data therefore reveal that *Dmrt1* supports SSC maintenance in two ways: allowing SSCs to remain in the stem cell pool under normal conditions; and enabling progenitor cells to help restore the stem cell pool after germ cell depletion.

## Author Summary

The *Dmrt1* gene is a deeply conserved gonadal regulator that is expressed in all mitotic germ cells of the mouse, including spermatogonial stem cells (SSCs). We previously showed that *Dmrt1* controls the mitosis/meiosis switch in differentiating mouse spermatogonia. Here we have examined the role of *Dmrt1* in undifferentiated spermatogonia and found that *Dmrt1* plays two crucial roles in sustaining the population of SSCs. First, *Dmrt1* is required to maintain the SSC pool during normal conditions: loss of *Dmrt1* in SSCs causes loss of the SSC maintenance factor PLZF and differentiation of SSCs. This result suggests that *Dmrt1* is necessary for SSC self-renewal. Second, *Dmrt1* is required to replenish SSCs after germ line depletion. We found that *Ngn3*-positive transit amplifying cells normally do not contribute to *Id4*-positive SSCs, but can do so when germ cells are chemically depleted by busulfan treatment. However, when *Dmrt1* is lost in committed progenitor cells the ability to replenish SSCs after cytotoxic stress is completely lost. Our results suggest that *Dmrt1* is important for SSC homeostasis and may provide new avenues for SSC manipulation.

## Introduction

Mammalian spermatogenesis begins at puberty and most mammals make sperm throughout much of adult life, relying on a pool of spermatogonial stem cells (SSCs) (reviewed in [1]). In the mouse, individual SSCs are found among the cohort of GFRα1-positive undifferentiated type A spermatogonia (A_undiff_). A_undiff_ occur as single cells (A_single_, or A_s_), connected pairs (A_paired_, or A_pr_) or chains of 4 to 16 cells (A_aligned_, or A_al_) formed by incomplete cytokinesis [1,2]. Differentiation begins when A_al_ cells transition to c-KIT-positive A_1_ spermatogonia [3]. A_1_ spermatogonia subsequently undergo five additional rounds of amplifying mitotic divisions accompanied by further differentiation, producing A_2_, A_3_, A_4_, Intermediate (In), and type B spermatogonia. The type B spermatogonia divide and differentiate into preleptotene spermatocytes that undergo meiosis [1].

SSC maintenance requires somatic niche factors including GDNF, which is produced by Sertoli cells and signals through the SSC cell surface receptors RET and GFRα1 [4]. Loss of *Gdnf* or either of its coreceptors *Ret* and *Gfra1* causes SSC depletion, while overexpression of GDNF causes accumulation of undifferentiated A_s_ cells [4–6]. SSC maintenance also is controlled by intrinsic factors including the transcriptional regulator PLZF, whose loss causes a progressive failure of spermatogenesis [7,8].

The precise identity of the SSC pool is still being established. The original SSC model, known as the A_s_ model, proposed that A_s_ cells are definitive stem cells and that formation of chains reflects commitment to differentiation [1,9]. However, in recent years, the A_s_ model has been challenged and refined by approaches including detailed expression analysis and live imaging. It is now clear that the A_s_ population is heterogeneous, with only a subset of A_s_ cells normally functioning as SSCs [2,10–14]. In addition, two major pools of A_undiff_ cells can be distinguished by the expression GFRα1 and NGN3. The GFRα1-positive population contains the great majority of SSC activity [11,12], while the NGN3-positive population normally functions as a pool of transit-amplifying cells that will eventually undergo differentiation and meiosis [15].

Recently, the transcriptional regulator ID4 was shown to be expressed in a small subset of undifferentiated spermatogonia that closely correlate with SSC activity in functional assays, such as transplantation [12,16,17]. However, the pool of GFRα1-positive cells that includes the SSCs is dynamic. Lineage tracing and live imaging experiments showed that A_pr_ and A_al_ chains can fragment to generate A_s_ cells and shorter chains that are proposed to function as SSCs [2]. Moreover, even NGN3-positive spermatogonia, which normally will proceed to differentiation and meiosis, can form SSCs when the germ line is challenged by stresses such as cytotoxic busulfan treatment or transplantation [2,10]. Thus while much SSC activity resides in ID4-positive cells, cell fate commitment in the early spermatogonial lineage is surprisingly fluid. How the interconversion of undifferentiated spermatogonial cell types is regulated to achieve homeostasis and steady state spermatogenesis is yet to be established.

DMRT1 is a gonad-specific transcription factor related to the invertebrate sexual regulators Doublesex and MAB-3 and plays a key role in both germline and somatic development in the testis [18]. DMRT1 is expressed in spermatogonia but not in meiotic or postmeiotic germ cells [19]. DMRT1 has at least three distinct functions in male germ cell development in mice. First, during late fetal development on sensitive strain backgrounds DMRT1 acts as a tumor suppressor that promotes mitotic arrest and silences pluripotency genes including *Sox2*. *Dmrt1* mutant germ cells form testicular teratomas with high incidence in mice of a susceptible strain background [20] and GWAS studies linking DMRT1 to human germ cell cancer suggest that DMRT1 may act analogously in human germ cells [21]. Second, DMRT1 is required perinatally for reactivation of mitosis and migration of prospermatogonia to the stem cell niche and for their subsequent survival [22,23]. Third, DMRT1 regulates the mitosis/meiosis decision during adult steady state spermatogenesis: DMRT1 in NGN3-positive progenitor cells promotes spermatogonial proliferation and differentiation and inhibits premature meiotic initiation [24]. In addition to these germ line functions, DMRT1 is required to prevent testicular Sertoli cells from transdifferentiating into their ovarian equivalents, granulosa cells [25,26].

In this study we used conditional gene targeting to investigate whether *Dmrt1* plays a role in maintaining a functional SSC population. We found that loss of *Dmrt1* in putative SSCs caused progressive loss of spermatogenesis that was associated with reduced PLZF expression and loss of ID4- and GFRα1-positive spermatogonia. We also investigated the role of DMRT1 in recovery from spermatogonial depletion and found that DMRT1 is required in *Ngn3-Cre* expressing spermatogonia for the repopulation of the Id4-GFP-positive spermatogonia after their depletion by cytotoxic busulfan treatment. Our results therefore suggest that DMRT1 plays a dual role in SSC homeostasis, promoting both maintenance and replenishment of the SSC pool. In addition, our data demonstrate that *Ngn3*-positive spermatogonia can give rise to Id4-GFP positive cells and repopulate the SSC pool under conditions of stress. Based on its diverse and context-dependent functions, we propose a model in which DMRT1 functions as an essential partner for more specialized regulators of male gametogenesis.

## Results

### *Dmrt1*-mutant SSCs can undergo differentiation

**Fig 1:**
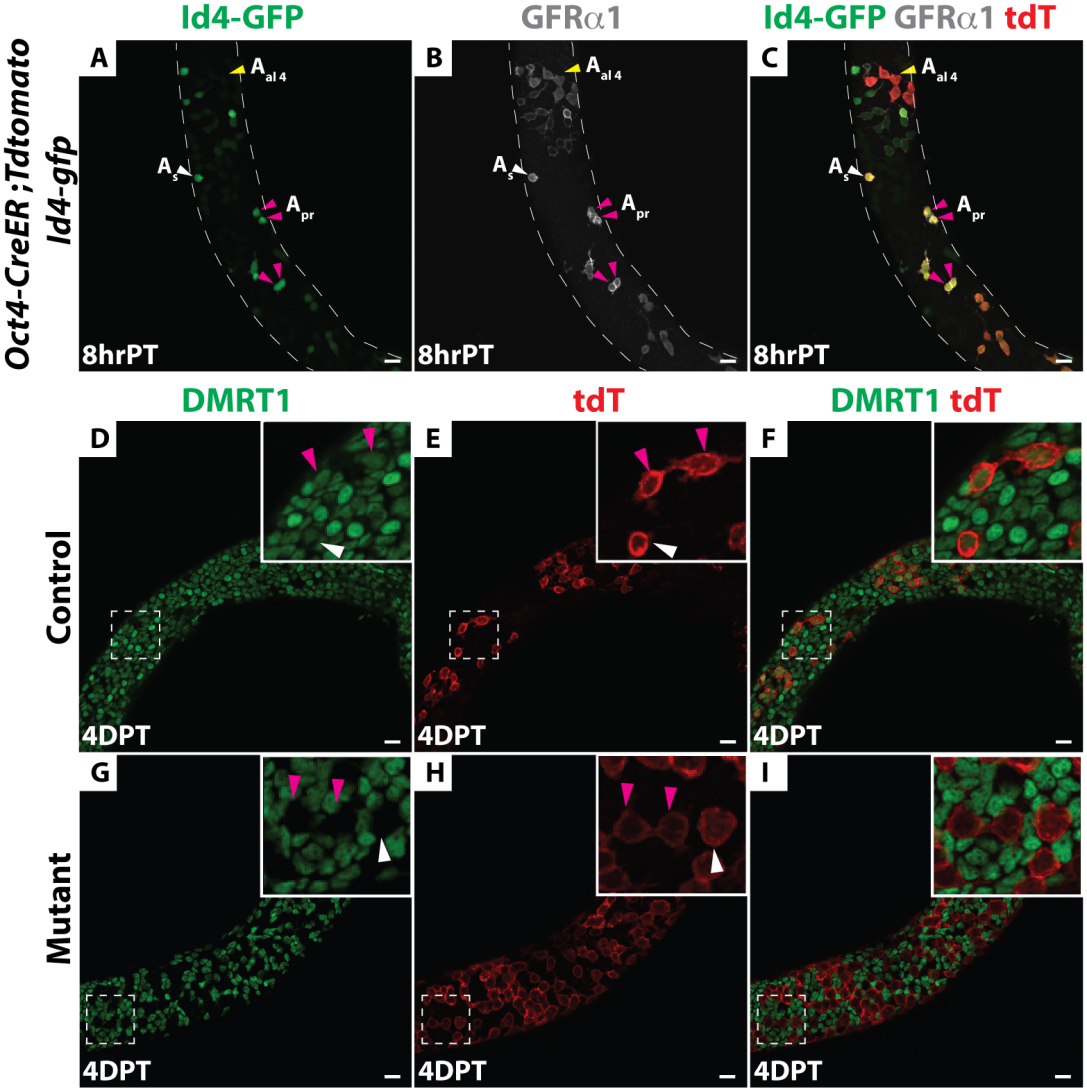
*Oct4-CreER* is active in SSCs and efficiently deletes *Dmrt1*. (*A-C*) Immunofluorescence (IF) of wholemount seminiferous tubules. *Oct4-CreER; Rosa26-tdTomato; Id4-Gfp* testes eight hours (hr) post-tamoxifen (PT) treatment. (*A*) GFP (green). (*B*) GFRα1 (gray). (*C*) Merge with RFP (red). Anti-RFP labels all tdTomato-positive cells (labeled “tdT” here and in subsequent figures). A_s_ spermatogonia are indicated by white arrowheads, A_pr_ are indicated by magenta arrowheads and A_al_ are indicated by yellow arrowheads. (*D-I*) IF of wholemount seminiferous tubules of *Dmrt1*^*flox*^*/+;Oct4-cre/+; tdTomato*; *Id4-Gfp* (hereafter, “control”) mice to the same strain homozygous for *Dmrt1*^*flox*^ (hereafter, “mutant”) stained for DMRT1 (green) and tdTomato (red) four days post-tamoxifen (DPT). In the control (*D-F*) DMRT1 protein expressed in tdTomato-positive A_s_, A_pr_ (inset) and A_al_ cells. In mutant (*G-I*) DMRT1 is depleted from tdTomato-positive A_s_, A_pr_ (inset) and A_al_ cells. Scale bars: 20 μm.

DMRT1 is expressed throughout spermatogonial development, in all A_s_ through type B spermatogonia, and then it is silenced in preleptotene spermatocytes at the onset of meiosis [19,24]. To confirm DMRT1 expression in SSCs, we performed immunofluorescence (IF) on wholemount seminiferous tubules from mice carrying a *Id4-Gfp* transgene [12], using anti-DMRT1 and anti-SALL4 antibodies. All A_s_ and A_pr_ cells expressing Id4-GFP also were positive for DMRT1 and SALL4 (S1A–S1M Fig). To test the role of DMRT1 in SSCs, we sought to conditionally delete *Dmrt1*. Because A_s_ and short-chain undifferentiated spermatogonia express *Oct3/4* [27] we tested a tamoxifen-inducible *Oct4-CreER* transgene [28] for activity in SSCs. To identify SSCs and detect *Cre* activity, respectively, we included *Id4-Gfp* and CRE-responsive *Rosa26-tdTomato* transgenes [16]. Mice were treated with tamoxifen at postnatal day 8, when SSCs are abundant [17] and the mechanistically distinct first wave of spermatogenesis has already initiated [15, 29]; testes were analyzed eight hours later. IF on wholemount seminiferous tubules using an anti-RFP antibody to detect tdTomato showed that many tdTomato-positive A_s_ and A_pr_ cells co-expressed Id4-GFP and GFRα1 and thus were putative SSCs (Fig 1A–1C). We also detected short chains of tdTomato-positive A_al_ cells that expressed GFRα1; some were weakly positive and some negative for Id4-GFP (Fig 1A–1C). We conclude that *Oct4-Cre* is active in GFRα1-positive undifferentiated spermatogonia including some of the *Id4-Gfp* positive SSCs.

**Fig 2:**
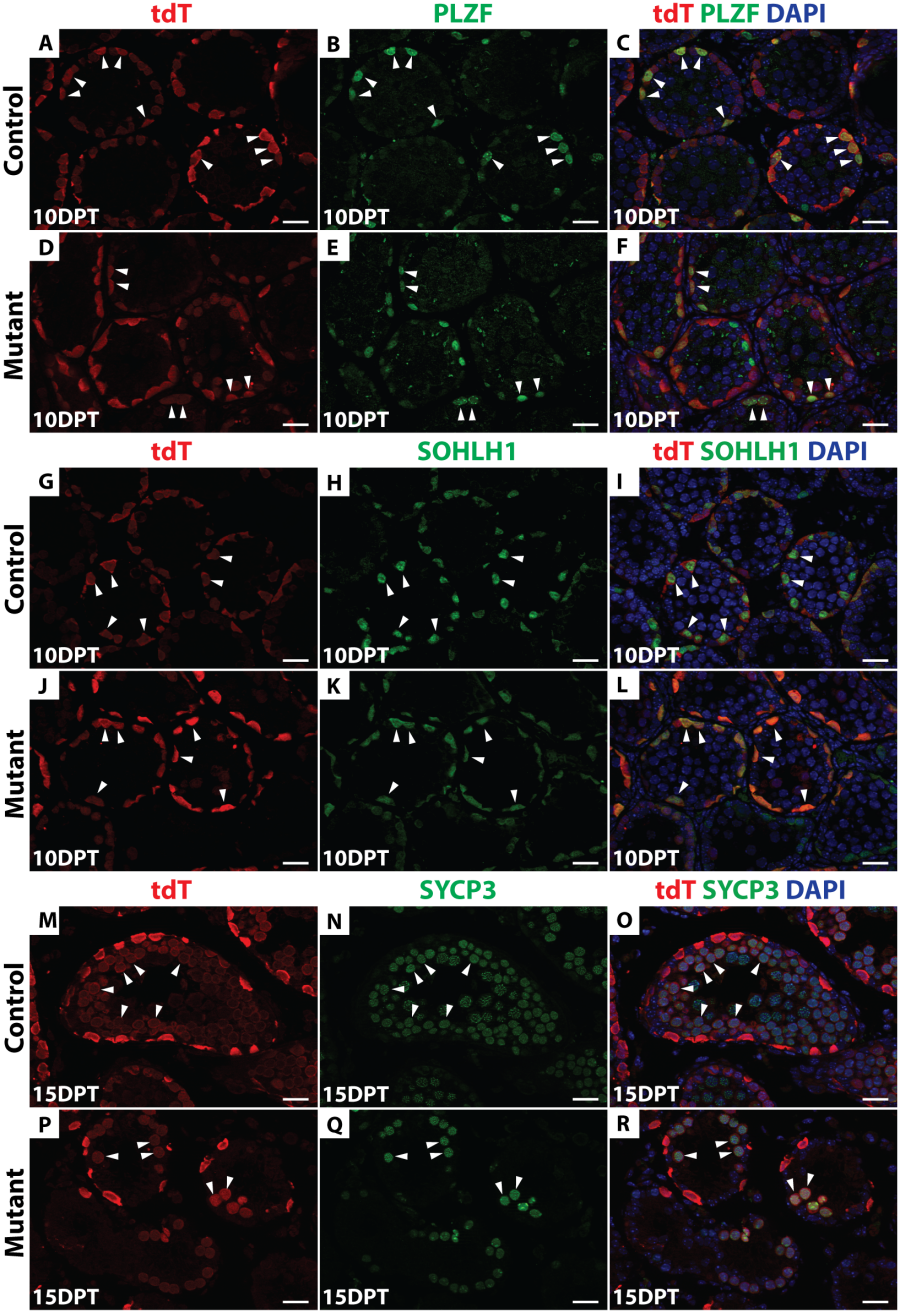
*Dmrt1* mutant undifferentiated spermatogonia can differentiate. IF of sectioned control (*A-C*, *G-I*, *M-O*) and conditional mutant *(D-F*, *J-L*, *P-R)* seminiferous tubules 10 and 15 days post-tamoxifen (DPT) detecting tdTomato (red) and three stage-specific germ cell markers (green). DAPI (blue) stains DNA. Anti-PLZF (*A-F*) was used to detect undifferentiated spermatogonia, anti-SOHLH1 (*G-L*) for differentiating spermatogonia and anti-SYCP3 (*M-R*) for meiotic cells. Double-positive cells are labeled by white arrowheads. Scale bars: 20μm.

Next we used *Oct4-Cre* to delete *Dmrt1*, comparing *Dmrt1*^*flox*^*/+;Oct4-cre/+; tdTomato*; *Id4-Gfp* (hereafter, “control”) mice to the same strain homozygous for *Dmrt1*^*flox*^ (hereafter, “mutant”). Cre recombination of *Dmrt1*^*flox*^ removes the proximal promoter and exon 1, which contains the DNA binding domain, generating a null allele of *Dmrt1* [30]. Four days post-tamoxifen injection (4 DPT), IF confirmed that DMRT1 was absent from tdTomato-positive A_s_ and A_pr_ as well as A_al_ cells (Fig 1D–1I). To follow the fates of undifferentiated tdTomato-labeled control and *Dmrt1* mutant spermatogonia, we traced these cells using IF on sectioned tubules to detect tdTomato and cell type-specific markers, including PLZF (undifferentiated spermatogonia), SOHLH1 (differentiating spermatogonia) and SYCP3 (primary spermatocytes). At 10 DPT, tdTomato-positive cells expressing PLZF or SOHLH1 were present in both control and mutant testes (Fig 2A–2L), indicating that in mutants, as in controls, a proportion of undifferentiated spermatogonia could progress to SOHLH1-positive differentiating spermatogonia. At 15 DPT, tdTomato-positive cells expressing SYCP3 were observed in both the control and mutant testes, indicating that *Dmrt1* mutant spermatogonia were able to enter meiosis (Fig 2M–2R). The reduced number of SYCP3-positive cells in mutants is consistent with our previous finding that *Dmrt1*-mutant undifferentiated spermatogonia can enter meiosis but they do so precociously, without completing the normal number of mitotic divisions [24]. Taken together, these data suggest that DMRT1 is not required for differentiation of SSCs or for initiation of meiotic prophase.

### DMRT1 is required for SSC maintenance

**Fig 3:**
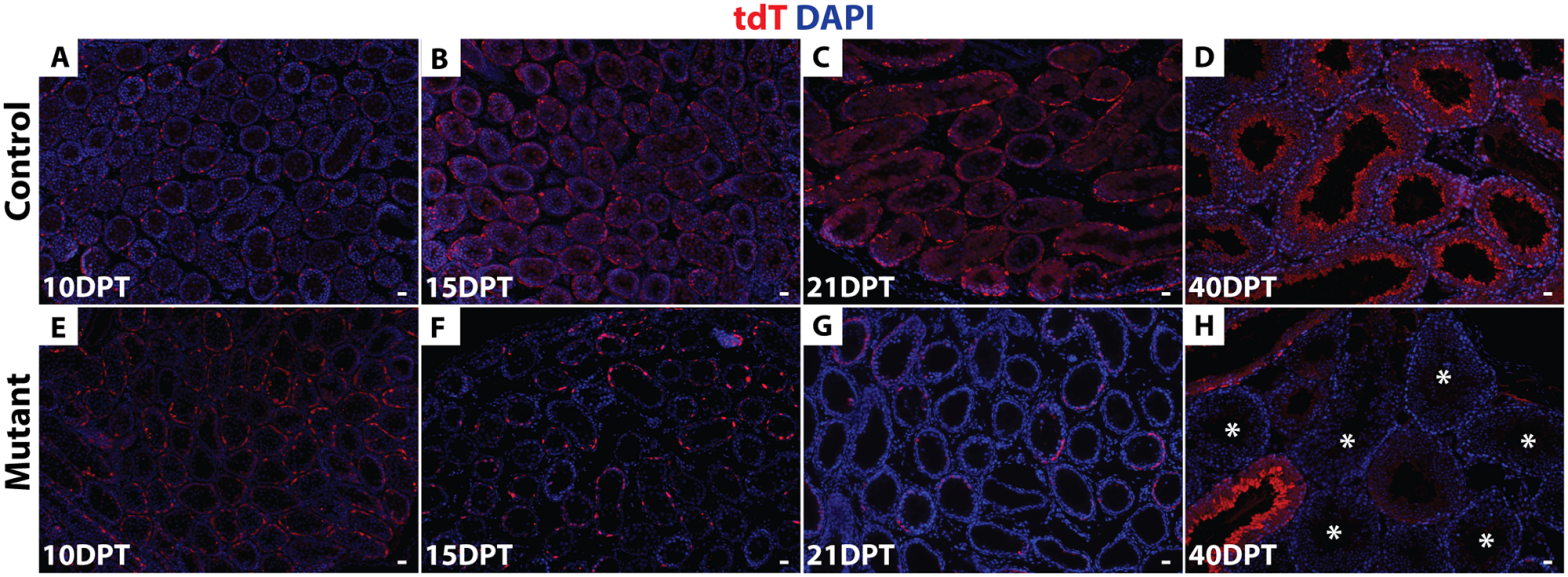
*Dmrt1* is required in undifferentiated spermatogonia to maintain spermatogenesis. (*A-H*) IF of sectioned testes showing tdTomato (red) at 10, 15, 21 and 40 DPT. DAPI DNA stain (blue). In controls (*A-D*) tdTomato-positive cells are maintained for a full round of spermatogenesis (40d). In mutant testes (*E-H*) tdTomato-positive cells were progressively lost, with more than 60% of tubules (N = 101/167) having no tdTomato-positive cells by 40 DPT (indicated by asterisks). Scale bars: 20 μm.

Next, we asked whether deletion of *Dmrt1* in A_s_, A_pr_ and A_al_ cells impairs SSC maintenance, which would be expected to cause a progressive loss of mutant germ cells. We followed tdTomato-positive cells for a full round of spermatogenesis (approximately 40 days) starting at 10 DPT. tdTomato-positive spermatogonia and early spermatocytes were present at 10 DPT (Fig 3A and 3E) in both control and mutant testes. In controls, at 15, 21 and 40 DPT, tdTomato-positive late spermatocytes, round and elongated spermatids were present (Fig 3B–3D). Importantly, all tubules were labeled with tdTomato and all germ cell types became tdTomato-positive in controls by 40 DPT, indicating that spermatogenesis was maintained via tdTomato-positive SSCs. In mutant testes, by contrast, tdTomato-positive cells were completely absent from ~40% of tubules at 21 DPT (N = 85/213), and from ~60% by 40 DPT (N = 101/167) (Fig 3G and 3H). After 16 DPT, spermatogonia that persisted in mutant testes expressed DMRT1, indicating that they had escaped deletion by *Oct4-cre*. Collectively these results indicate that deletion of *Dmrt1* in A_s_, A_pr_ and A_al_ cells causes progressive loss of spermatogenesis that affects all germ cell types.

**Fig 4:**
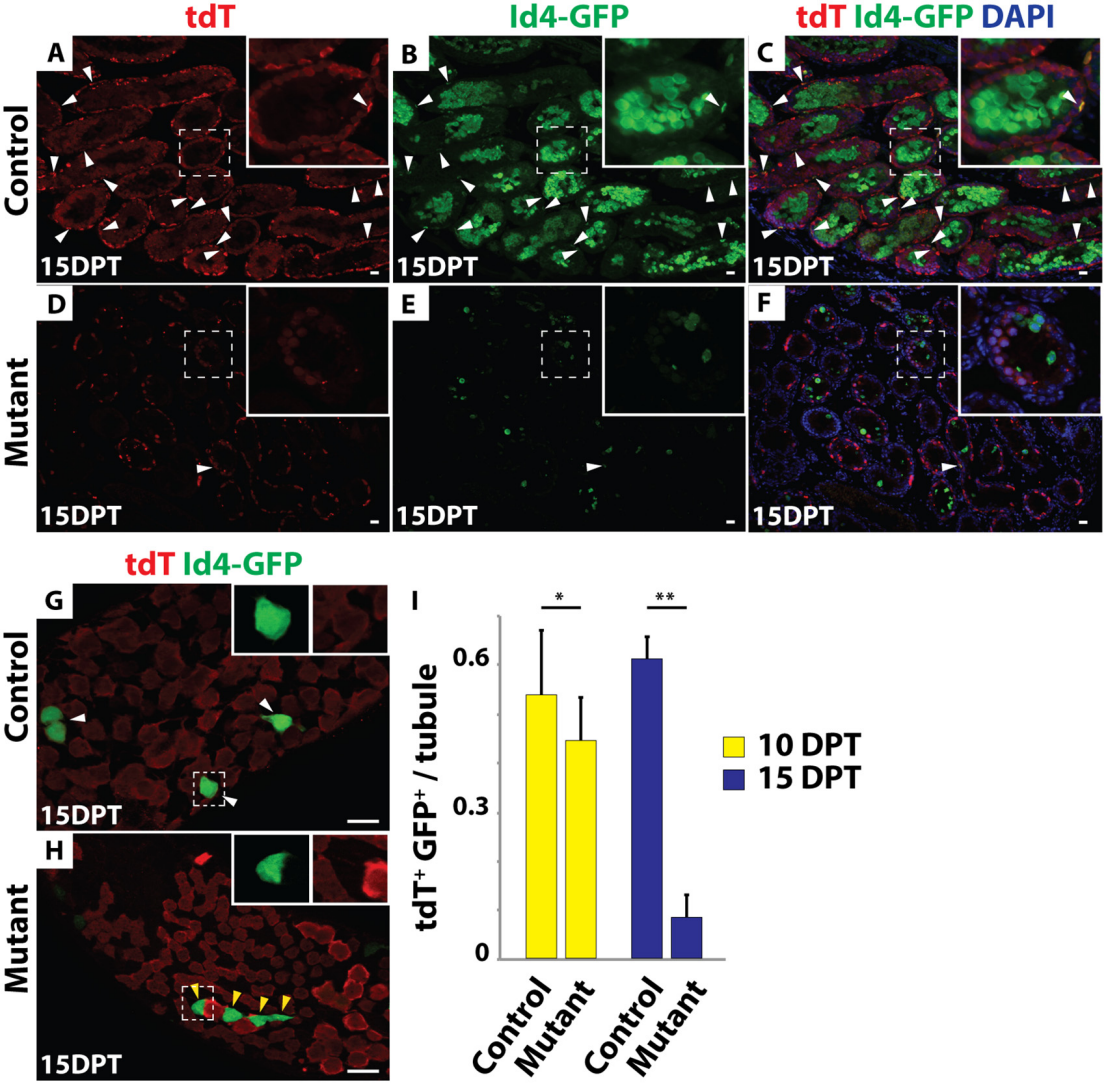
*Dmrt1* mutant SSCs are not maintained. (*A-F*) IF of sectioned 15 DPT testes showing tdTomato (red) and GFP (green). DAPI DNA stain (blue). All tdTomato/GFP double-positive cells are labeled with white arrowheads. In control testes (*A-C*) many tdTomato/GFP double-positive cells were present, while in mutant testes (*D-F*) double-positive cells were rare. Higher magnifications are shown in insets. GFP-positive meiotic cells represent previously described ectopic expression of the *Id4-Gfp* transgene (11). (*G*, *H*) IF of wholemount seminiferous tubules 15 DPT showing tdTomato (red) and GFP (green). In control (*G*) all tdTomato-positive cells were GFP-positive (white arrowhead; inset), while in mutant (*H*), GFP-positive cells almost all lacked tdTomato (yellow arrowhead; inset) indicating that they were wild-type. Scale bars: 20μm. (*I*) Quantification of Id4-GFP and tdTomato double-positive cells in control and mutant testes 10 (yellow) and 15 (blue) DPT. Values are average from >300 tubules. Error bars indicate standard deviation. * indicates P < 0.05 (Student’s T test). ** indicates P<0.005.

We more closely followed the fate of mutant germ cells by examining expression of Id4-GFP, GFRα1 and tdTomato at 10 and 15 DPT. Many Id4-GFP and tdTomato double-positive cells were present in the control testes at both time points (Fig 4A–4C and 4I) but double-positive cells were decreased in the mutants by 10 DPT and were rarely observed in the mutants by 15 DPT (N>300 tubule sections) (Fig 4D–4F and 4I). Similarly, IF of wholemount seminiferous tubules revealed that the remaining Id4-GFP- and GFRα1-positive cells in mutant testes were virtually all negative for tdTomato (Fig 4G and 4H; S2A–S2D Fig), indicating that they had escaped inactivation of *Dmrt1*. From these results we conclude that deletion of *Dmrt1* in A_s_, A_pr_ and A_al_ cells causes a failure to maintain SSCs.

**Fig 5:**
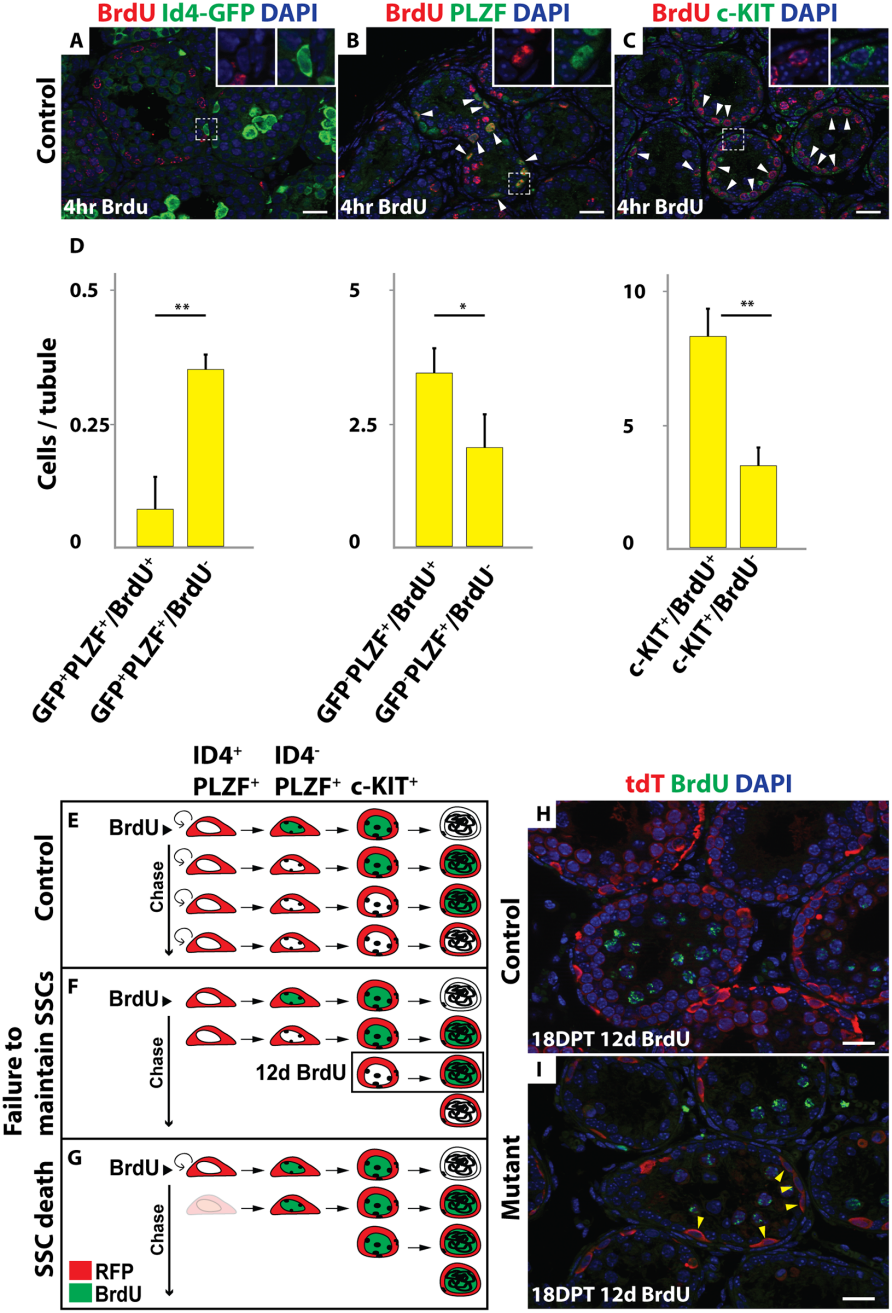
Mutant SSCs are lost due to defective maintenance. (*A-C*) Controls treated with BrdU for 4 hours. IF of sectioned testes showing BrdU (red) and spermatogonial markers: Id4-GFP, PLZF or c-KIT (green). DAPI DNA stain (blue). Double-positive cells are indicated by the white arrowhead. Higher magnifications are shown in insets. *(D)* Quantification of BrdU and GFP^+^/PLZF^+^; GFP^-^/PLZF^+^ and c-KIT^+^ positive cells. Values are average from >300 tubule sections for GFP staining, and >100 for PLZF and c-KIT staining. Error bars indicate standard deviation. ** indicates P < 0.005 (Student’s T test). *(E-G)* Diagram of the BrdU chase experiment in the control *(E)* and two possible outcomes in the mutant: failure to maintain SSCs *(F)* and SSC death *(G)*. *(H*, *I)* IF of sectioned testes showing tdTomato (red) and BrdU (green) in control (*H*) and mutant (*I*) 18 DPT with BrdU chase for 12 days. DAPI DNA stain (blue). Double-positive cells are indicated by the white arrowheads and tdTomato single-positive cells are indicated by yellow arrowheads. Scale bars: 20 μm.

We next investigated how SSCs are lost in mutants. TUNEL labeling at 10 and 15 DPT and activated Caspase3 staining at 12 DPT showed no apparent increase in mutants and did not detect apoptosis of Id4-GFP positive cells, suggesting that loss of spermatogonia in mutants is not due to elevated apoptosis (S3A–S3J Fig). However, apoptosis in SSCs might be infrequent and difficult to detect. We therefore examined the dynamics of spermatogenesis using lineage tracing and BrdU labeling. Specifically, we asked whether SSCs that were tdTomato-labeled by *Oct4-Cre* continue to contribute to spermatogenesis after *Dmrt1* is deleted. In controls, Id4-GFP-positive cells were only rarely BrdU-labeled (15.3%; N>300 tubule sections); however Id4-GFP negative spermatogonia that were PLZF-positive or c-KIT-positive efficiently incorporated BrdU (61.9% and 70.8%, respectively; N>100 for each staining) (Fig 5A–5D). This differential labeling provides a means to distinguish between fates of SSCs versus other spermatogonial cell types. Next we injected BrdU at 6 DPT (N = 3 for each genotype), when SSCs were still present in mutant testes, and compared controls and mutants after a 12-day chase. In controls, the SSCs produced a population of BrdU-negative spermatogonia that replaced the differentiating BrdU-positive cells (Fig 5E and 5H). In mutants, we anticipated one of two possible outcomes after the chase. If the BrdU-negative SSCs survived but were not maintained, they would produce a transient population of BrdU-negative spermatogonia that would eventually enter meiosis (Fig 5F). Alternatively, if the BrdU-negative mutant SSCs died, no further BrdU-negative spermatogonia would be formed and remaining germ cells would be primarily BrdU-positive (Fig 5G). We observed the former result (Fig 5I), with many tdTomato-positive (*Dmrt1* mutant) but BrdU-negative spermatogonia present for at least 18 days after the chase. Because some Id4-GFP negative spermatogonia were BrdU negative this experiment is not definitive. However, based on the lack of detectable apoptosis and the prolonged presence of BrdU negative differentiating spermatogonia, we conclude that the loss of SSCs in *Dmrt1* conditional mutants likely is due mainly to failed SSC maintenance/self-renewal rather than cell death.

### DMRT1 promotes PLZF expression in A_undiff_ spermatogonia

**Fig 6:**
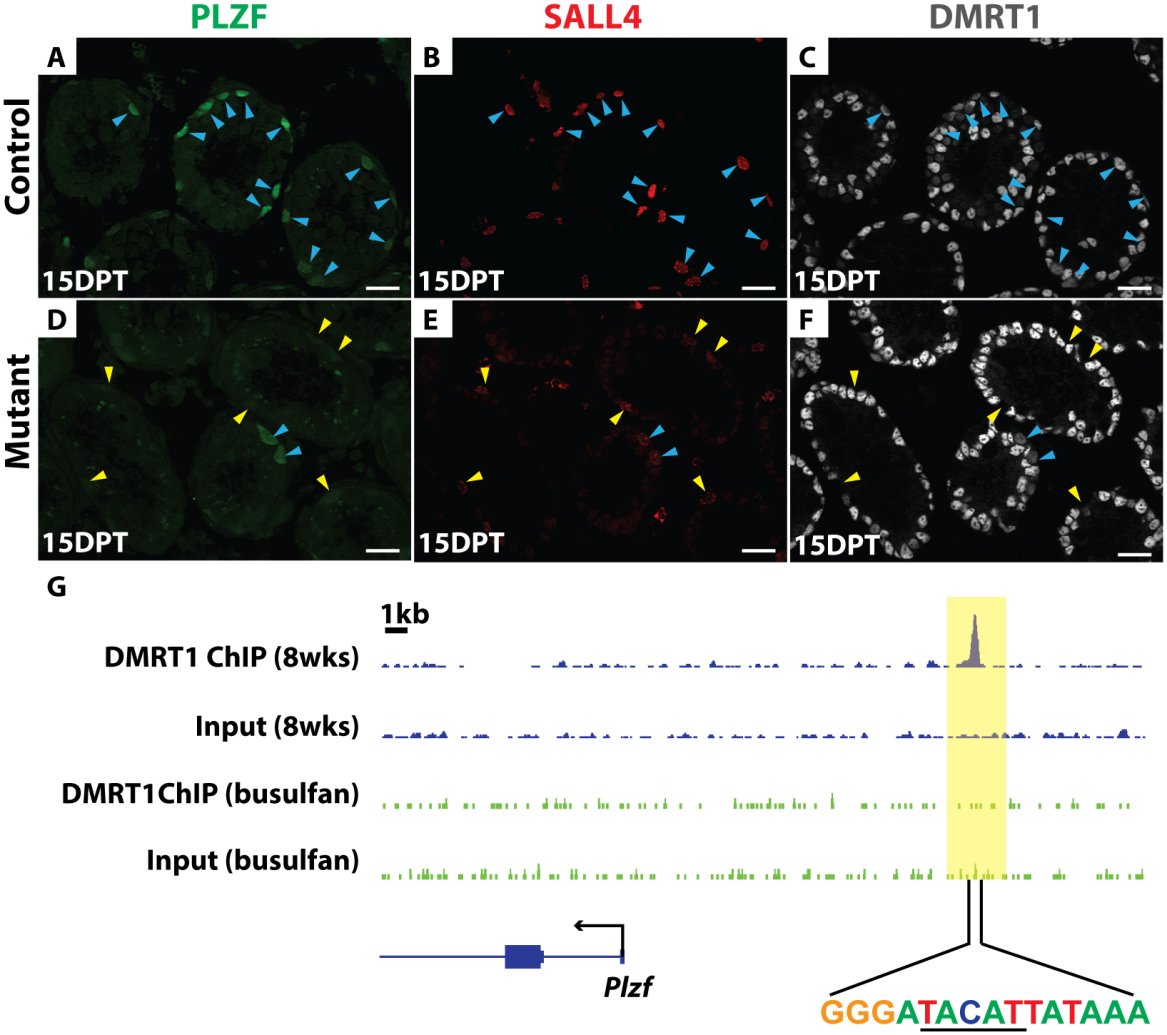
PLZF expression requires DMRT1. *(A-F)* IF of sectioned testes showing PLZF (green), SALL4 (red), and DMRT1 (gray) staining in controls (*A-C)* and mutant (*D-F)* at 15 DPT. DAPI DNA stain (blue). DMRT1-positive germ cells are indicated by blue arrowheads, and DMRT1-negative germ cells by yellow arrowheads. DMRT1-negative and SALL4-positive germ cells consistently have reduced PLZF relative to DMRT1-positive cells. Scale bars: 20μm. *(G)* ChIP-seq showing that DMRT1 binds near *Plzf*. Wild-type adult (blue track) and adult testes treated with 30 mg/kg busulfan for 4-weeks (green track) to deplete germ cells were analyzed. The data range shown for each binding profile is 0 to 2 read counts/million. DNA sequence centered under the binding site is shown, with a DMRT1 consensus half-site underlined.

We next investigated the molecular basis of SSC loss in *Dmrt1* mutants. A key regulator of SSC maintenance is the transcription factor PLZF, which is expressed in undifferentiated spermatogonia. In *Plzf* mutants spermatogenesis fails after one round in some tubules [7], similar to the phenotype of conditional *Dmrt1* mutants described above. All PLZF-positive spermatogonia normally express DMRT1 and SALL4 (Fig 6A–6C). IF analysis of *Dmrt1* mutant testes indicated that PLZF expression was severely reduced in DMRT1-negative and SALL4-positive mutant spermatogonia relative to nearby SALL4 and DMRT1-positive cells that escaped deletion by *Oct4-cre* (Fig 6D–6F). Although we cannot distinguish which of the PLZF/SALL4 double-positive cells are SSCs in this experiment, all SALL4-positive cells without DMRT1 had low PLZF expression. We therefore conclude that one function of DMRT1 in undifferentiated spermatogonia is to maintain PLZF expression, and reduced PLZF is likely to be a key contributor to SSC loss in *Dmrt1* mutants.

ChIP-Seq in intact adult testes showed that DMRT1 binds near *Plzf* (Fig 6G). Because DMRT1 is expressed both in spermatogonia and Sertoli cells we used a high dose of busulfan (30 mg/kg) to deplete germ cells and repeated the ChIP-seq, asking whether binding was reduced in the absence of germ cells. Indeed, DMRT1 binding was substantially reduced in busulfan-treated testes. Although we cannot distinguish in which spermatogonial cells DMRT1 binds *Plzf*, this result indicates that most of the binding is germ cell-dependent and therefore DMRT1 may directly activate *Plzf* in spermatogonia (Fig 6G). The DNA sequence centered under the DMRT1 binding peak contained a DMRT1 consensus half-site rather than the canonical palindromic sequence element (Fig 6G) [31,32]. The lack of a complete canonical DMRT1 binding sequence suggests that DMRT1 might bind with a non-DMRT partner protein at this site.

### *Dmrt1* is required in *Ngn3*-positive spermatogonia for SSC replenishment

During steady state spermatogenesis, NGN3 expression marks a transition from strong SSC potential to committed transit-amplifying cells that normally proliferate and differentiate, eventually entering meiosis. Lineage tracing using *Ngn3-Cre* has shown that NGN3-positive cells only rarely function as SSCs during steady state, but some of them can be induced to form stable SSCs by stresses including transplantation or germ cell chemical depletion using busulfan [2,10,19]. Thus NGN3-positive spermatogonia have been suggested to provide a reserve stem cell pool for times of stress [10].

Because *Dmrt1* is required perinatally to establish SSCs [23] and subsequently for SSC maintenance (this work), we asked whether it also plays a role in replenishment of SSCs from NGN3-positive spermatogonia in response to cytotoxic stress. We used a moderate dose of busulfan (20 mg/kg) to deplete most undifferentiated spermatogonia [33] and tested whether loss of *Dmrt1* in *Ngn3-Cre* expressing cells compromises SSC regeneration. To follow cell fates we again used *Rosa26-tdTomato* as a lineage tracer and employed Id4-GFP to identify putative SSCs.

We first confirmed that in adult mice under steady-state conditions *Ngn3-Cre* expressing cells rarely contribute to the pool of Id4-GFP positive SSCs. In controls, tdTomato expression activated in *Ngn3-Cre*-positive undifferentiated spermatogonia could be traced through to elongated spermatids but we detected no tdTomato in Id4-GFP-positive SSCs (S4A Fig). In *Dmrt1* conditional mutants, the number of differentiating spermatogonia was severely reduced by precocious meiotic initiation that occurs upon loss of *Dmrt1* in NGN3-positive spermatogonia [24]. As in controls, there was no tdTomato labeling of Id4-GFP-positive spermatogonia (N>200 tubules for each genotype) (S4B Fig). Thus, in both wild-type and mutant testes, *Ngn3*-positive spermatogonia normally proceed to meiosis and do not form Id4-GFP-positive SSCs.

We next followed the fate of *Ngn3-cre* positive cells during recovery after busulfan injection. Four-week-old mice were injected with busulfan and examined 60 days post-injection, which should allow full restoration of spermatogenesis [33]. To confirm that Id4-GFP positive cells were lost after busulfan treatment, we quantified the number of Id4-GFP positive cells in cross-sections from untreated and treated control and mutant testes at 7, 10 and 20 days after busulfan injection. In the untreated control and mutant testes, we detected 1.7 and 1.4 Id4-GFP positive cells per tubule cross-section, respectively (N>400), indicating that starting SSC populations were similar. In controls at 7 days post injection we detected 0.11 Id4-GFP positive cells per tubule and <0.001 at 10 and 20 days (N>300) (S5 Fig). Similarly, in mutant testes at 7 days we detected 0.07 Id4-GFP cells per tubule and <0.001 at 10 and 20 days post injection, respectively (N>300) (S5 Fig).

**Fig 7:**
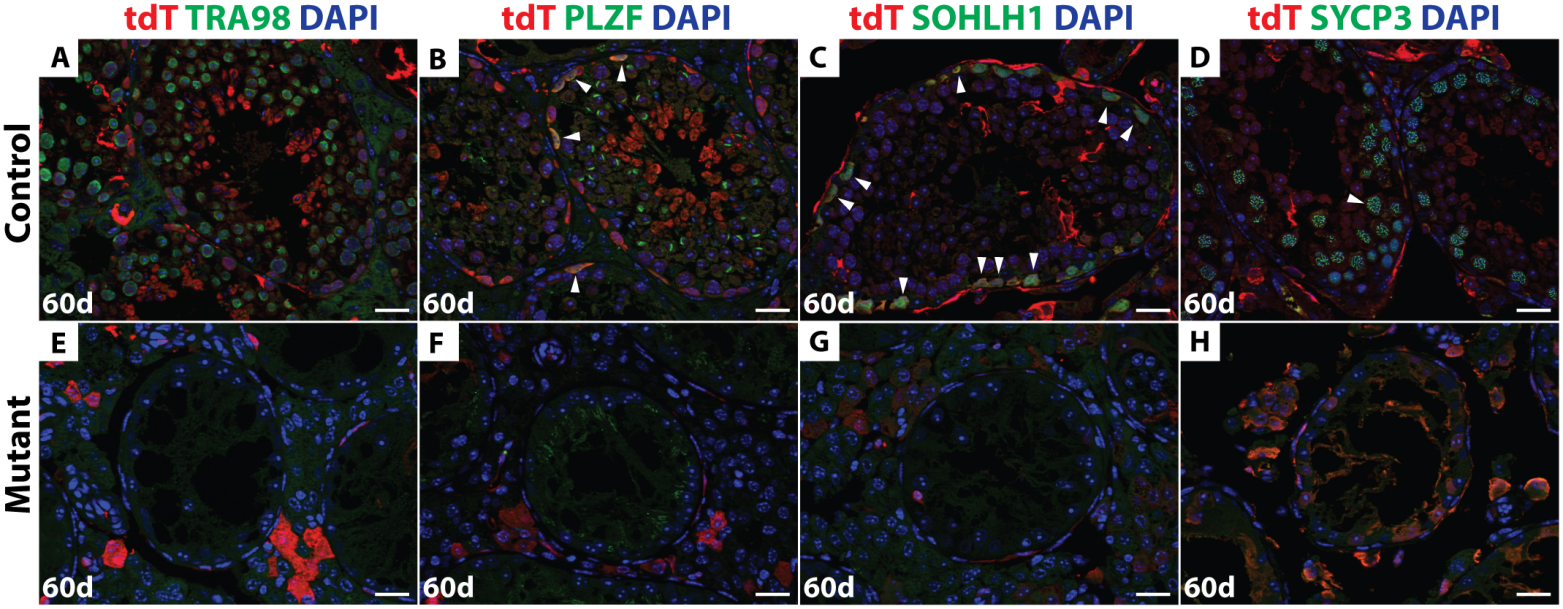
*Dmrt1* is required for the recovery of spermatogenesis post-busulfan treatment. IF of sectioned testes 60 days after busulfan treatment double stained with tdTomato (red) and TRA98, PLZF, SOHLH1 or SYCP3 (green). DAPI DNA stain (blue). In the control (*A-D*) all stages of germ cells that are tdTomato-positive were detected (white arrowhead). In the mutant (*E-H*), no germ cells were observed. Scale bars: 20 μm.

**Fig 8:**
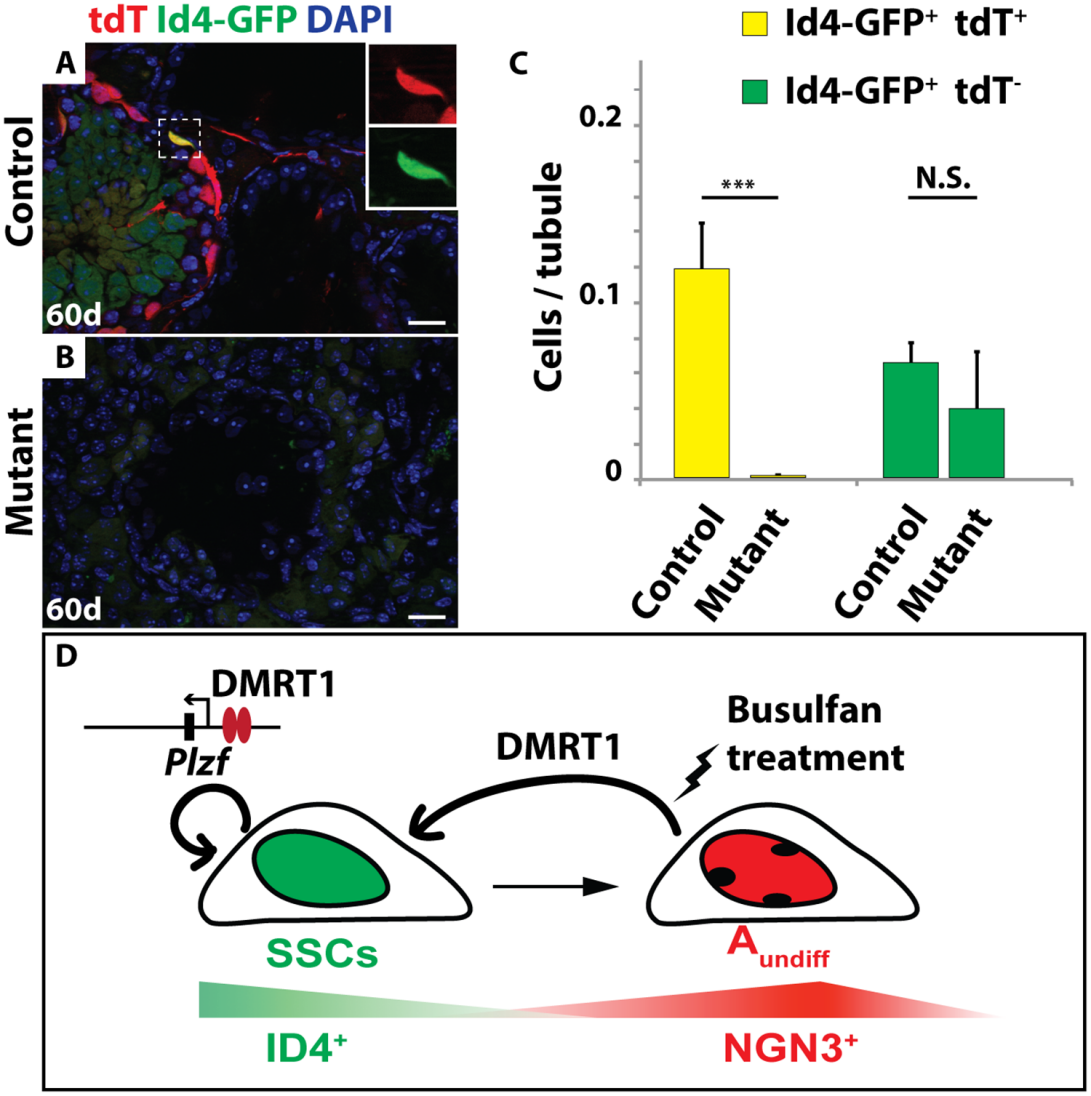
*Dmrt1* is required for SSC regeneration. IF of sectioned adult testes showing tdTomato (red) and GFP (green) 60 days after busulfan treatment (20mg/kg). DAPI DNA stain (blue). In *Dmrt1*^*flox*^*/+; Ngn3-cre/+; tdTomato*; *Id4-Gfp* control testes (*A*) tdTomato and GFP double-positive cells were observed after 60 days (inset) but in *Dmrt1*^*flox/flox*^*; Ngn3-cre/+; tdTomato; Id4-Gfp* mutant (*B*) no double-positive cells were observed. Scale bars: 20μm. (*C*) Quantification of Id4-GFP and Id4-GFP/tdTomato double-positive cells in control and mutant testes. Double-positive cells are shown in yellow, GFP-positive cells are shown in green. Values are average from >200 tubule sections. Error bars indicate standard deviation. *** indicates P < 0.0005 (Student’s T test). (D) Model of DMRT1 during SSC homeostasis. SSCs are indicated in green, NGN3-positive undifferentiated spermatogonia are indicated in red.

By 60 days post-busulfan treatment, controls had recovered abundant TRA98-expressing germ cells and spermatogenesis appeared normal in 68% of seminiferous tubules (146/216) (Fig 7A). All major stages of spermatogonia and spermatocytes were present after recovery, as reflected by expression of PLZF, SOHLH1 and SYCP3 (Fig 7B–7D). The majority of germ cells in the recovered testes of busulfan-treated control mice, including the Id4-GFP-positive putative SSCs, expressed tdTomato (Figs 7A–7D and 8A), indicating that they were derived from *Ngn3-Cre*-positive spermatogonia. In contrast, 60 days post-busulfan treatment, 95% of mutant seminiferous tubules (177/187) were completely devoid of TRA98-positive germ cells and lacked Id4-GFP, PLZF-, and SOHLH1-positive spermatogonia along with SYCP3-positive primary spermatocytes (Figs 7E–7H and 8B). While Id4-GFP-positive cells were present in mutants after recovery, all of these were negative for tdTomato, indicating that they had not passed through an *Ngn3*-positive stage and were not derived from *Dmrt1* mutant cells (Fig 8C).

A simple interpretation of our results is that replenishment of SSCs from *Ngn3*-positive cells requires *Dmrt1*. However, we considered two other possibilities. First, loss of *Dmrt1* may render spermatogonia more susceptible to busulfan treatment, and thus fewer undifferentiated spermatogonia might survive to help replenish spermatogenesis in mutants. To test this possibility, we counted the number of PLZF and SALL4 double-positive undifferentiated spermatogonia at 7, 10 and 20 days post busulfan treatment in control and mutant mice. At the two earlier time points the number of PLZF/SALL4-positive spermatogonia was similar between controls and mutants (S5 Fig), indicating that undifferentiated spermatogonia were not unusually busulfan-sensitive in the mutants. At 20 days PLZF/SALL4-positive cells were starting to recover in controls but not in mutants. Second, NGN3-positive cells might be able to revert back to SSCs in the mutant but, due to lack of DMRT1, they would not be maintained and would differentiate. However, there was no evidence of an additional wave of spermatogenesis derived from such cells. Therefore a likely model is that DMRT1 is required in *Ngn3*-positive spermatogonia for their re-expression of ID4 and re-establishment as SSCs.

## Discussion

Here we have found that *Dmrt1* plays two roles, both of which are expected to support SSC homeostasis, as diagrammed in Fig 8D. First, *Dmrt1* is required within the SSC pool for efficient maintenance, with mutant SSCs losing PLZF expression and undergoing differentiation, resulting in a progressive loss of spermatogenesis. Second, under cytotoxic stress, *Dmrt1* is required in NGN3-positive spermatogonia for renewal of the SSC pool.

*In vivo* lineage tracing and apoptosis assays together suggested that *Dmrt1* mutant SSCs do not undergo apoptosis but instead at least some of them lose PLZF expression and undergo differentiation. The resulting deficiency in SSC self-renewal causes a gradual failure of spermatogenesis. While our data suggest that transcriptional activation of *Plzf* by DMRT1 may be a key component of SSC maintenance, it will be important to identify additional targets of DMRT1 regulation in this cell type. Our results suggesting survival and differentiation of *Dmrt1* mutant SSCs contrast with an *in vitro* study that found depletion of *Dmrt1* can cause apoptosis of cultured multipotent germline stem cells [34]. The different behavior of *Dmrt1* mutant germ cells in these two studies might reflect differences between the stem cell microenvironment *in vivo* and *in vitro* or differences in the specific cell types studied. Given the findings here and those of prior *in vivo* studies suggesting that DMRT1 function in germ cells is highly context-dependent, different requirements for *Dmrt1 in vivo* and in cell culture are perhaps unsurprising.

Although recovery from busulfan germ cell depletion was documented decades ago [33], the genetic basis of the process is uncertain. Yoshida and colleagues [35] showed that some NGN3-positive cells can provide SSC function after damage or transplantation. It has been unclear whether damage or transplantation triggers the regeneration of normal SSCs or perhaps instead they trigger formation of a less primitive self-renewing cell population, analogous to that found in the hematopoetic cell lineage [36]. Our lineage tracing data indicate that NGN3-positive cells can give rise to Id4-GFP-positive cells after busulfan-induced cytotoxic stress, suggesting that normal SSCs are restored. These data are in good agreement with a recent study that demonstrated the recovery of ID4-positive cells after busulfan treatment but did not address the source of the new SSCs [17]. The possibility that transit-amplifying germ cells could undergo conditional reversion to SSC function was proposed previously and is consistent with our data [37,38]. In this regard SSC recovery may operate analogously to the conditional germ cell regeneration system found in the *Drosophila* testis [39–41]. However, while lineage tracing shows that the replenished Id4-GFP positive SSCs pass through an *Ngn3*-positive state, it is important to note that we cannot yet resolve the detailed path by which SSCs are reestablished. The simplest model is that SSCs are replenished by activating latent stem cell potential in the NGN3-positive transit-amplifying cells that normally supply steady-state spermatogenesis. However, it also remains possible that SSCs are not killed by busulfan, but instead they transiently activate *Ngn3* in response to germ cell depletion. In such a scenario, the disappearance of Id4-GFP positive cells that we observed after busulfan treatment might reflect a temporary switch from *Id4* to *Ngn3* expression. Distinguishing between these possibilities will require lineage tracing of ID4-positive cells after busulfan treatment. Also, while lineage tracing showed that *Ngn3*-positive cells contribute to SSC replenishment, we cannot exclude that *Ngn3*-negative spermatogonia also can contribute.

Regardless of the initial source of the replenishing spermatogonia, our data show that replenishment of Id4-GFP positive SSCs requires DMRT1 activity and involves NGN3-positive spermatogonia, since loss of *Dmrt1* in NGN3-positive germ cells led to a complete failure to recover from busulfan treatment. We previously found that loss of *Dmrt1* in NGN3-positive spermatogonia during steady state spermatogenesis in essence flips a switch, truncating the spermatogonial differentiation and mitotic proliferation program and sending spermatogonia toward meiosis [24]. DMRT1 promotes spermatogonial proliferation and differentiation by suppressing retinoic acid (RA) signaling and transcriptionally repressing the RA target *Stra8* [42]. It is possible that DMRT1 acts similarly in replenishment of SSCs: mutant cells may be forced to proceed toward meiosis and thus unable to form SSCs, or they may be unable to remain undifferentiated long enough to reactivate the SSC program. The reduced expression of PLZF in *Dmrt1*-mutant undifferentiated spermatogonia also seems likely to limit SSC replenishment, either because SSCs cannot be formed or because they cannot be maintained once they are reestablished.

Our findings add to a surprisingly long list of essential roles for DMRT1 in germ cells and somatic cells of the gonad. The multiplicity of its functions and their context-dependence strongly suggest that DMRT1 cannot be a purely instructive regulatory factor and must serve a permissive role in some contexts. This context-dependence is particularly clear in spermatogonial development, where DMRT1 promotes maintenance of SSCs, promotes differentiation of NGN3-positive cells during steady state spermatogenesis, and promotes SSC regeneration after germ cell depletion. While some of these functions are likely to be mechanistically related, it seems likely that DMRT1 functionally interacts with other more specialized factors to accomplish these distinct roles. In this view DMRT1 would be an essential partner for regulators that act in specific cell types or physiological conditions. Working with different partners could allow different regulatory outcomes at particular sites under different conditions or could allow recognition of non-canonical DNA sequences, for example the sequence bound by DMRT1 upstream of *Plzf*. Identifying these hypothetical partners and defining their functions should be informative.

In summary, our findings indicate that DMRT1 is an important regulator of SSC homeostasis, acting in two distinct roles. Under normal conditions we suggest that DMRT1 promotes SSC maintenance at least in part by regulating self-renewal via activation of *Plzf* transcription. After busulfan-induced cytotoxic stress, DMRT1 enables *Ngn3*-positive germ cells to replenish the SSC pool and restore spermatogenesis. These findings provide a basis to further explore SSC homeostasis and may have relevance to male infertility.

## Materials and Methods

### Animals

*Dmrt1*^*flox/flox*^ mice were bred to either *Oct4-CreERT2* [28] (gift of Dr. Yoav Segal) or *Ngn3-Cre* [29]; (gift of Dr. Shosei Yoshida) and to *Id4-Gfp* [12] and *Rosa26-tdTomato* transgenic mice (Jackson Laboratories Cat #: 0007914). For experiments involving conditional deletion of *Dmrt1*, *Dmrt1*^*flox/+*^ mice were used as controls and *Dmrt1*^*flox/flox*^ mice were used as experimentals. Both controls and experimental animals carried the *Oct4-cre* transgene and were treated with tamoxifen. In busulfan depletion experiments, controls and experimental animals carried the *Ngn3-cre* transgene and were either *Dmrt1*^*flox/+*^ (control) or *Dmrt1*^*flox/flox*^ (experimental). Experimental protocols were approved by the University of Minnesota Institutional Animal Care and Use Committee.

### Immunofluorescent of testis tissue sections

Immunofluorescence (IF) was described [43]. Staining was performed on >5 animals from each genotype. Antibodies are listed in S1 Table. Expression of tdTomato from the cre activity reporter transgene was detected using an anti-RFP antibody.

### Immunofluorescent of whole mount seminiferous tubules

Whole mount IF was described [35]. All whole mount images were captured with a LSM 710 confocal. Staining was performed on >5 animals from each genotype. Antibodies used are listed in S1 Table.

### BrdU incorporation and TUNEL assay

BrdU incorporation and TUNEL assay were as described [24].

### Busulfan administration

Busulfan administration was described [35] except that we used 4-week-old male mice and concentration of 20 mg/kg.

### Tamoxifen injection

4-Hydroxy-tamoxifen injection was described [24] except that mice were injected at postnatal day 8 mice.

### ChIP-seq

Chromatin from testes of adult wild-type B6 and 129Sv mixed genetic background mice was cross-linked with formaldehyde, sheared, and immunoprecipitated with anti-DMRT1 antibody as described previously [44].

## Supporting Information

S1 Fig Id4-GFP positive A_s_ and A_pr_ express DMRT1. *(A-M)* IF of whole mount seminiferous tubules from mice carrying *Id4-Gfp* transgene using anti-DMRT1 (gray) and anti-SALL4 (red) antibodies. Id4-GFP positive cells are shown in green. Triple-positive cells are indicated by white arrowheads. Scale bars: 20μm.
(TIF)

S2 Fig *Dmrt1*-mutant GFRα1-positive spermatogonia are lost. (*A-D*) IF of whole mount seminiferous tubules at 15 DPT showing tdTomato (red) and GFRα1 (green). In control (*A* and *B*) tdTomato-positive cells were positive for GFRα1 (white arrowhead), while in mutant (*C* and *D*) the remaining GFRα1-positive cells were negative for tdTomato (yellow arrowhead) indicating that they were wild-type. Scale bars: 20μm.
(TIF)

S3 Fig Mutant SSCs are not lost due to apoptosis. (*A-D*) TUNEL assay detecting apoptosis (green) and DAPI DNA stain (blue). Comparing control (*A*, *C*) and mutant testes (*B*, *D*) sections at 10 and 15 DPT. (E-J) Activated Caspase3 IF detecting apoptosis (red), Id4-GFP IF (green) and DAPI DNA stain (blue). Arrows indicate GFP positive SSCs, which are negative for Caspase3. Scale bars: 20μm.
(TIF)

S4 Fig Contribution of NGN3-positive spermatogonia to SSCs is rare during steady state. IF of adult *Ngn3-cre; Rosa26-tdtomato; Id4-gfp* testes for tdTomato (red) and GFP labeling (green). DAPI DNA stain (blue). In control (*A*) Id4-GFP positive spermatogonia were negative for tdTomato (*inset*), indicating that NGN3-positive cells normally do not revert to ID4-positive cells. Similar results were observed in mutants (*B*). Background signals were observed in cells outside of the tubules. Scale bars: 20μm.
(TIF)

S5 Fig Busulfan sensitivity of mutant and wild type spermatogonia. Quantification of PLZF and SALL4 double-positive cells and Id4-GFP positive cells 7, 10 and 20 days after busulfan treatment (20mg/kg) in control and mutant testes. PLZF and SALL4 double-positive cells are shown in red, Id4-GFP positive cells are shown in green. Values are average from >200 tubules for each time point. Error bars indicate standard deviation. N.S. indicates the data is not statistically significant (Student’s T test), *** indicates P < 0.0005 (Student’s T test).
(TIF)

S1 Table Primary and secondary antibodies used for immunofluorescence. (DOCX)
