# Supplementary figures and images for: DMRT1 Is Required for Mouse Spermatogonial Stem Cell Maintenance and Replenishment
Source: PLoS Genet. 2016 Sep 1;12(9):e1006293. doi: 10.1371/journal.pgen.1006293 (PMC5008761; doi:10.1371/journal.pgen.1006293)

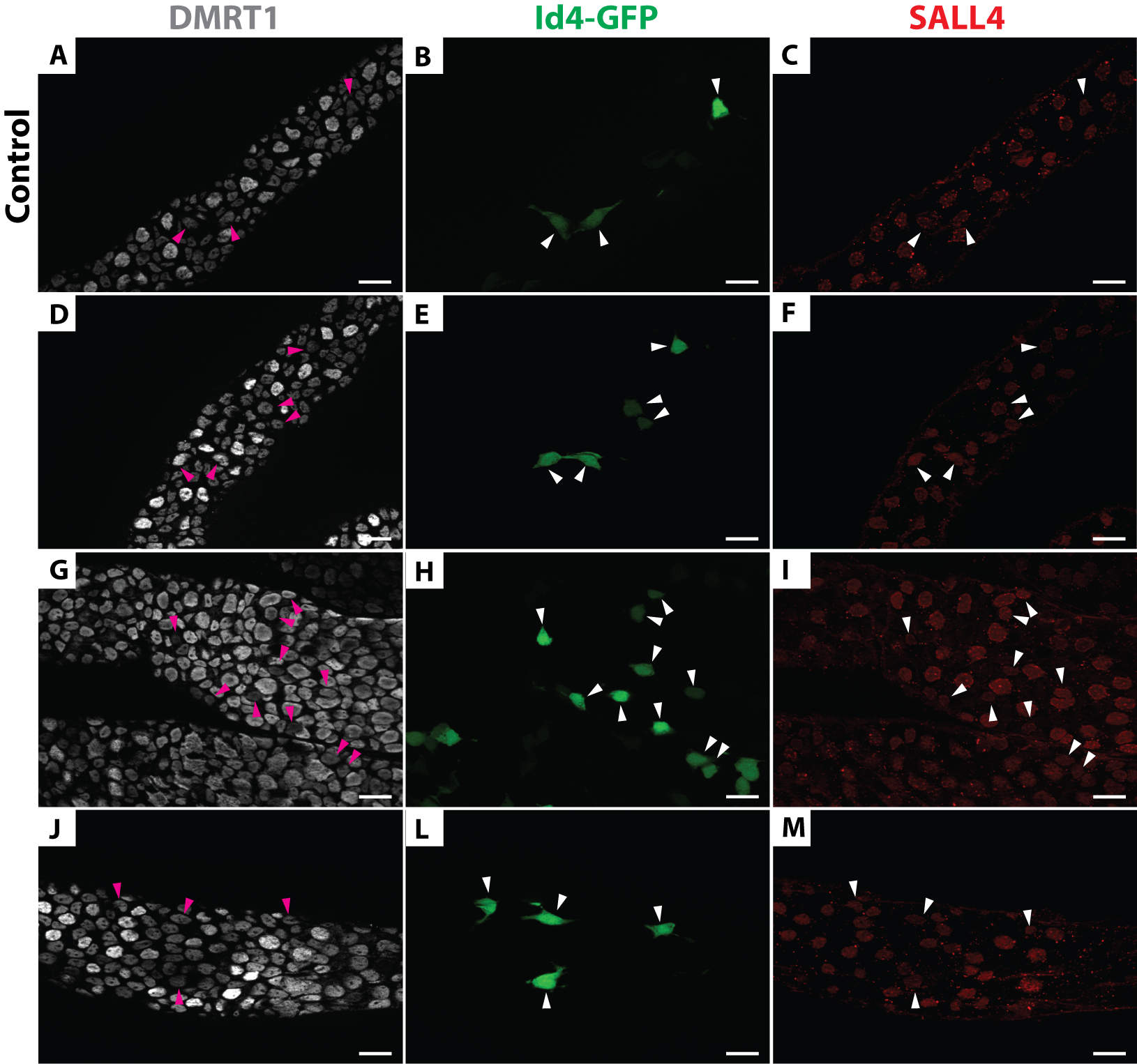

Supplement: S1 Fig — (A-M) IF of whole mount seminiferous tubules from mice carrying Id4-Gfp transgene using anti-DMRT1 (gray) and anti-SALL4 (red) antibodies. Id4-GFP positive cells are shown in green. Triple-positive cells are indicated by white arrowheads. Scale bars: 20μm. (TIF) [file pgen.1006293.s001.tif]

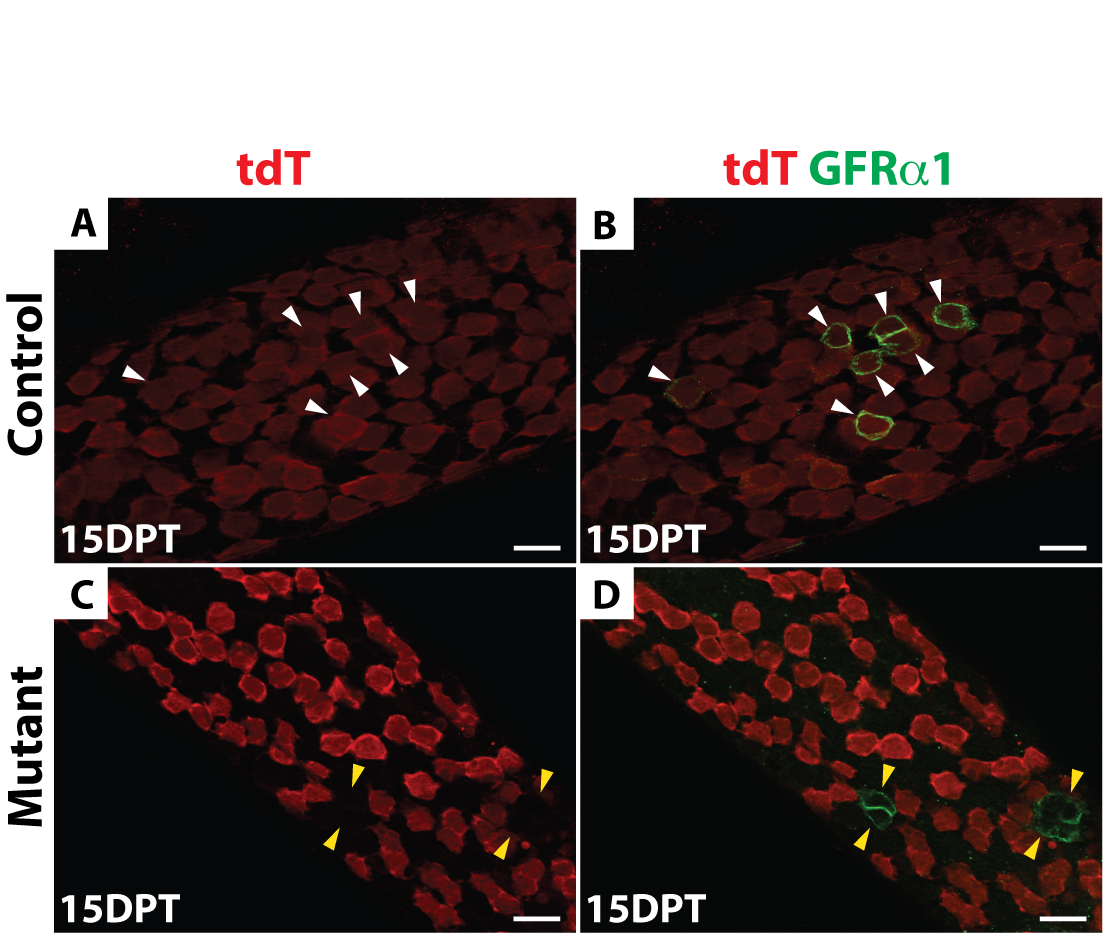

Supplement: S2 Fig — (A-D) IF of whole mount seminiferous tubules at 15 DPT showing tdTomato (red) and GFRα1 (green). In control (A and B) tdTomato-positive cells were positive for GFRα1 (white arrowhead), while in mutant (C and D) the remaining GFRα1-positive cells were negative for tdTomato (yellow arrowhead) indicating that they were wild-type. Scale bars: 20μm. (TIF) [file pgen.1006293.s002.tif]

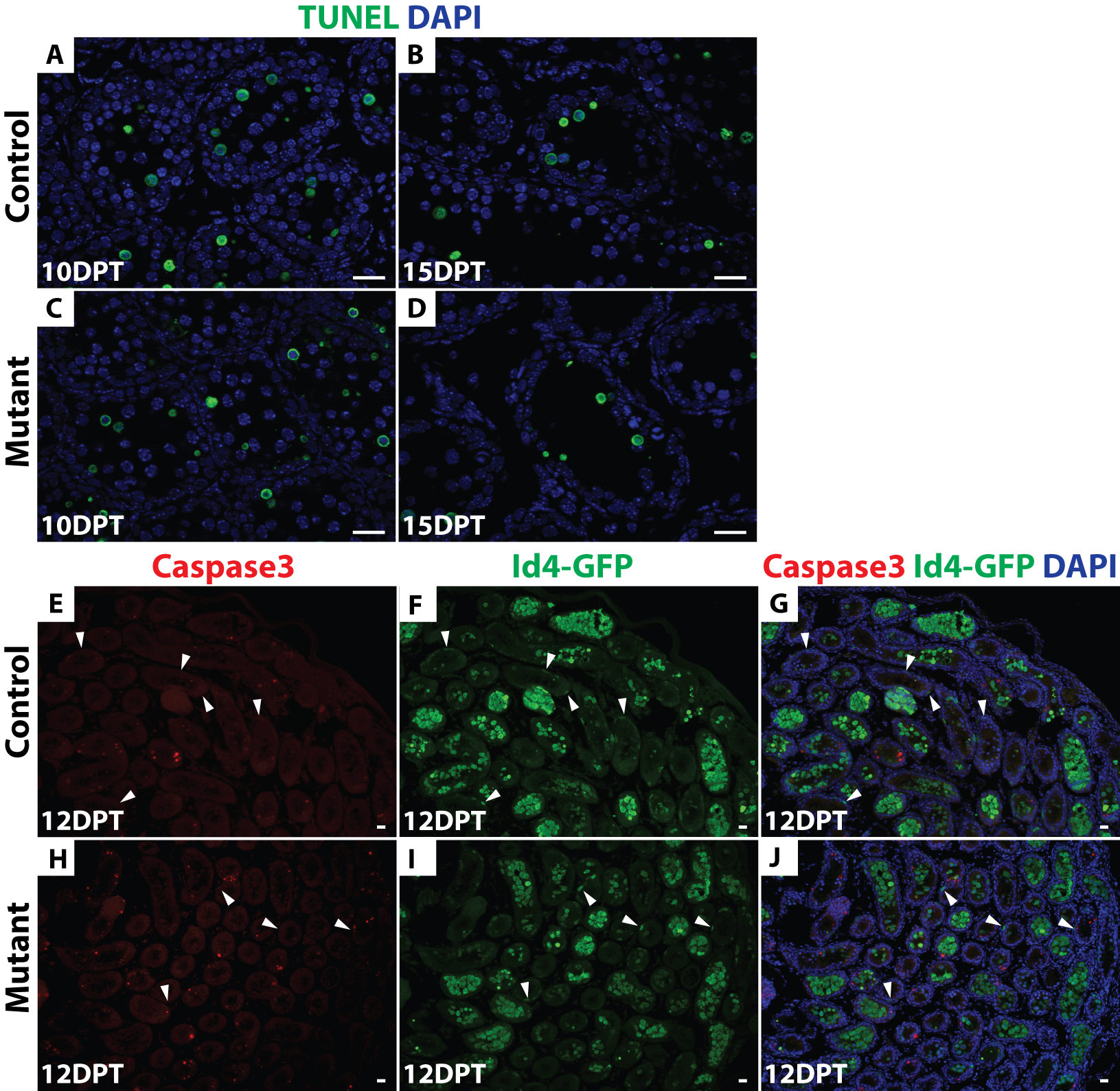

Supplement: S3 Fig — (A-D) TUNEL assay detecting apoptosis (green) and DAPI DNA stain (blue). Comparing control (A, C) and mutant testes (B, D) sections at 10 and 15 DPT. (E-J) Activated Caspase3 IF detecting apoptosis (red), Id4-GFP IF (green) and DAPI DNA stain (blue). Arrows indicate GFP positive SSCs, which are negative for Caspase3. Scale bars: 20μm. (TIF) [file pgen.1006293.s003.tif]

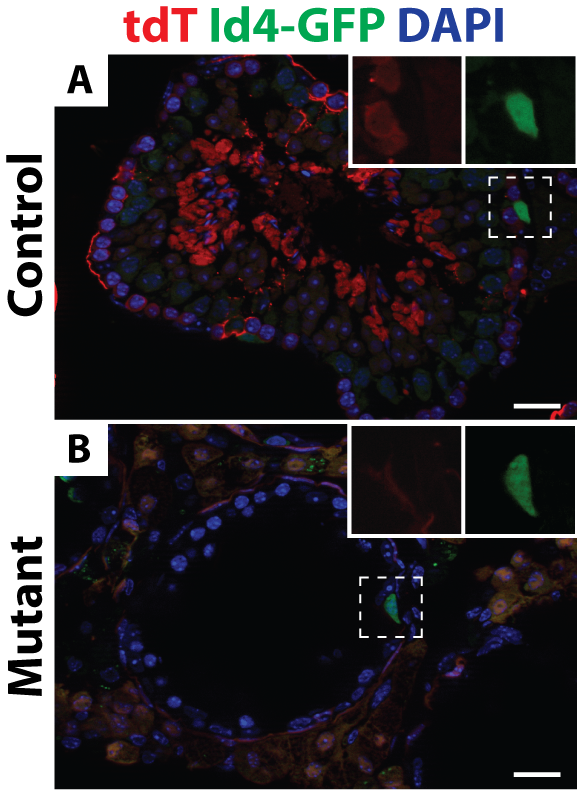

Supplement: S4 Fig — IF of adult Ngn3-cre; Rosa26-tdtomato; Id4-gfp testes for tdTomato (red) and GFP labeling (green). DAPI DNA stain (blue). In control (A) Id4-GFP positive spermatogonia were negative for tdTomato (inset), indicating that NGN3-positive cells normally do not revert to ID4-positive cells. Similar results were observed in mutants (B). Background signals were observed in cells outside of the tubules. Scale bars: 20μm. (TIF) [file pgen.1006293.s004.tif]

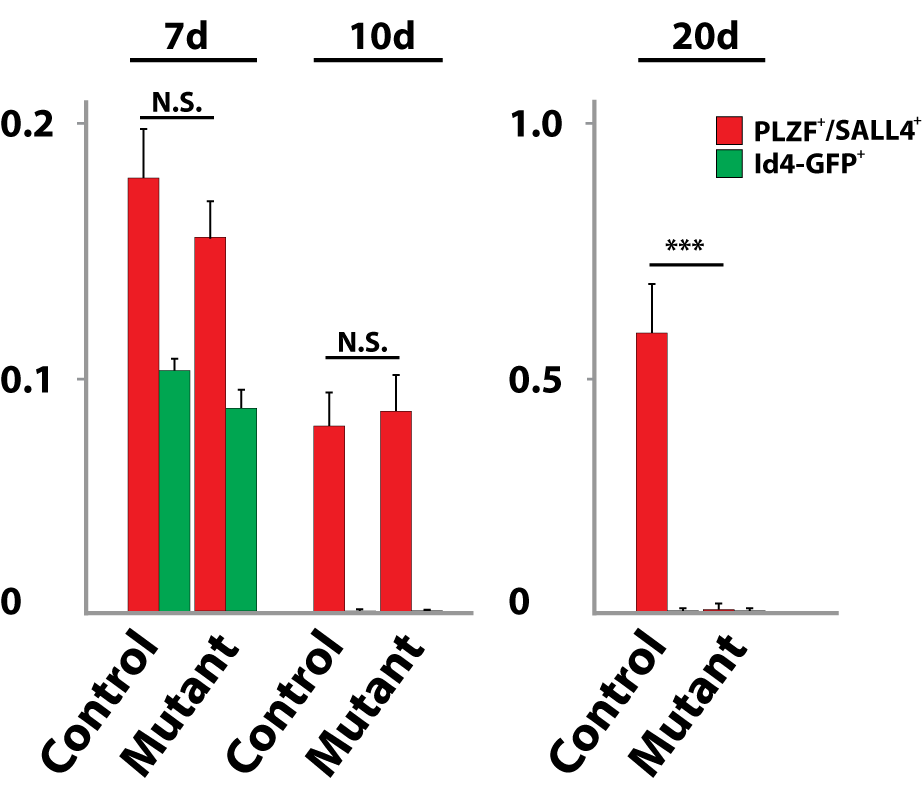

Supplement: S5 Fig — Quantification of PLZF and SALL4 double-positive cells and Id4-GFP positive cells 7, 10 and 20 days after busulfan treatment (20mg/kg) in control and mutant testes. PLZF and SALL4 double-positive cells are shown in red, Id4-GFP positive cells are shown in green. Values are average from >200 tubules for each time point. Error bars indicate standard deviation. N.S. indicates the data is not statistically significant (Student’s T test), *** indicates P < 0.0005 (Student’s T test). (TIF) [file pgen.1006293.s005.tif]
