# Supplementary material for: DMRT1 Is Required for Mouse Spermatogonial Stem Cell Maintenance and Replenishment
Source: PLoS Genet. 2016 Sep 1;12(9):e1006293. doi: 10.1371/journal.pgen.1006293 (PMC5008761; doi:10.1371/journal.pgen.1006293)
Supplement: S1 Table — (DOCX) [file pgen.1006293.s006.docx]

**S1. Table.** Primary and secondary antibodies used for immunofluorescence

| **Primary Antibody** |  | **Dilution** | **Source** | **Reference** |
| --- | --- | --- | --- | --- |
| **Brdu** | Rat monoclonal (IgG) | 1:200 | Abcam | Cat. No. ab6326 |
| **Caspase-3** | Rabbit polyclonal | 1:100 | Cell Signaling Technology | Cat No. 9662 |
| **c-KIT** | Rabbit polyclonal | 1:200 | Cell Signaling Technology | Cat No. 3074 |
| **DMRT1** | Rabbit polyclonal | 1:200 | David Zarkower | Raymond et al. 2000 PMID: 11040213 |
| **GFP** | Goat polyclonal | 1:500 | Novus Biologicals | Cat. No. AF4240 |
| **GFRa1** | Goat polyclonal | 1:50 | R&D Systems | Cat. No. AF560 |
| **PLZF** | Mouse monoclonal (IgG) | 1:200 | Calbiochem | Cat. No. OP128 |
| **RFP** | Rabbit polyclonal | 1:50 | Rockland | Cat. No. [600-401-379](https://www.rockland-inc.com/store/Antibodies-to-GFP-and-Antibodies-to-RFP-600-401-379-O4L_24299.aspx) |
| **SALL4** | Mouse monoclonal (IgG_1_) | 1:200 | Santa Cruz Biotechnology | Cat. No. [sc-101147 (EE-30)](https://www.rockland-inc.com/store/Antibodies-to-GFP-and-Antibodies-to-RFP-600-401-379-O4L_24299.aspx) |
| **SOHLH1** | Guinea pig polyclonal | 1:100 | Aleksandar Rajkovic | Pangas et al. PMID: 1472434 |
| **SYCP3** | Mouse polyclonal | 1:200 | Abcam | Cat. No. ab96672 |
| **TRA98** | Rat monoclonal (IgG) | 1:200 | Bio Academia | Cat. No. 73-003  PMID: 9568529 |

| **Secondary Antibody** |  | **Dilution** | **Source** | **Reference** |
| --- | --- | --- | --- | --- |
| **Anti Goat IgG**  **Alexa Fluor 594** | Donkey polyclonal | 1:500 | Invitrogen | Cat. No. A11058 |
| **Anti Goat IgG**  **Alexa Fluor 488** | Donkey polyclonal | 1:500 | Invitrogen | Cat. No. A11055 |
| **Anti Goat IgG**  **Alexa Fluor 647** | Donkey polyclonal | 1:500 | Invitrogen | Cat. No. A21447 |
| **Anti Rabbit IgG**  **Alexa Fluor 594** | Donkey polyclonal | 1:500 | Invitrogen | Cat. No. A21207 |
| **Anti Rabbit**  **Alexa Fluor 488** | Donkey polyclonal | 1:500 | Invitrogen | Cat. No. A21206 |
| **Anti Rabbit**  **Alexa Fluor 647** | Donkey polyclonal | 1:500 | Invitrogen | Cat. No. A31573 |
| **Anti Mouse**  **Alexa Fluor 594** | Donkey polyclonal | 1:500 | Invitrogen | Cat. No. A21203 |
| **Anti Rat**  **Alexa Fluor 488** | Donkey polyclonal | 1:500 | Invitrogen | Cat. No. A21208 |
| **Anti Guinea pig**  **Alexa Fluor 488** | Donkey polyclonal | 1:500 | Jackson ImmunoResearch Inc. | Cat. No. 706-545-148 |

­­
